# The Intermolecular Interaction of Ephexin4 Leads to Autoinhibition by Impeding Binding of RhoG

**DOI:** 10.3390/cells7110211

**Published:** 2018-11-15

**Authors:** Kwanhyeong Kim, Juyeon Lee, Hyunji Moon, Sang-Ah Lee, Deokhwan Kim, Susumin Yang, Dae-Hee Lee, Gwangrog Lee, Daeho Park

**Affiliations:** 1 School of Life Sciences and Aging Research Institute, Gwangju Institute of Science and Technology, Gwangju 61005, Korea; khkim0409@gist.ac.kr (K.K.); iris260@gist.ac.kr (J.L.); hjmoon311@gist.ac.kr (H.M.); sanga03@gist.ac.kr (S.-A.L.); po7322@gist.ac.kr (D.K.); susuminy@gist.ac.kr (S.Y.); gwangroglee@gist.ac.kr (G.L.); 2 Research Center for Cellular Homeostasis, Ewha Womans University, Seoul 03760, Korea; 3 Department of Oncology, College of Medicine, Korea University, Seoul 08308, Korea; neogene@korea.ac.kr

**Keywords:** Ephexin4, Ephexin, GEF, RhoG, autoinhibition, interaction

## Abstract

Ephexin4 is a guanine nucleotide-exchange factor (GEF) for RhoG and is involved in various RhoG-related cellular processes such as phagocytosis of apoptotic cells and migration of cancer cells. Ephexin4 forms an oligomer via an intermolecular interaction, and its GEF activity is increased in the presence of Elmo, an Ephexin4-interacting protein. However, it is uncertain if and how Ephexin4 is autoinhibited. Here, using an Ephexin4 mutant that abrogated the intermolecular interaction, we report that this interaction impeded binding of RhoG to Ephexin4 and thus inhibited RhoG activation. Mutation of the glutamate residue at position 295, which is a highly conserved residue located in the region of Ephexin4 required for the intermolecular interaction, to alanine (Ephexin4^E295A^) disrupted the intermolecular interaction and increased binding of RhoG, resulting in augmented RhoG activation. In addition, phagocytosis of apoptotic cells and formation of membrane ruffles were increased more by expression of Ephexin4^E295A^ than by expression of wild-type Ephexin4. Taken together, our data suggest that Ephexin4 is autoinhibited through its intermolecular interaction, which impedes binding of RhoG.

## 1. Introduction

The Rho family of small GTPases function as molecular switches for a variety of cellular events and are essential for cytoskeletal rearrangement and cell migration [1,2,3]. Rho GTPases are regulated by various guanine nucleotide-exchange factors (GEFs) and GTPase-activating proteins. GEFs catalyze the exchange of GDP for GTP in GTPases and thereby activate these enzymes. Conventional GEFs contain a tandem DH-PH domain and exchange GDP for GTP via the DH domain. Ephexins form a subfamily of conventional GEFs [4,5,6,7].

The Ephexin subfamily of GEFs has five members (Ephexin1–5) [8]. These proteins interact with EphA receptors and are thereby involved in various EphA receptor-mediated cellular processes. The function of Ephexin1 has been characterized relatively well. This protein is a GEF for RhoA and functions in axon guidance and spine morphogenesis by interacting with EphA4 [9,10,11,12]. Several other Ephexins are also GEFs for RhoA [13,14,15]; however, Ephexin4 displays GEF activity for RhoG, rather than for RhoA. Ephexin4 interacts with EphA2 and activates RhoG, which leads to cancer cell migration. We recently reported that Ephexin4 is also involved in phagocytosis of apoptotic cells and cooperates with Elmo via a biochemical interaction to activate RhoG, which leads to activation of Rac and promotes engulfment of apoptotic cells [8,16,17].

Ephexins contain a long N-terminus and a tandem DH–PH domain followed by a SH3 domain [18]. This suggests that Ephexins are structurally alike and that their GEF activity is regulated in a similar manner. Nevertheless, the molecular mechanism that regulates the activities of Ephexins remains elusive. A previous study by our group provides a clue to explain how Ephexin4 is regulated. Ephexin4 forms an oligomer via a homotypic intermolecular interaction mediated by its SH3 domain, and the GEF activity of Ephexin4 is increased in the presence of Elmo, which disrupts this interaction [19]. These findings suggest that the intermolecular interaction affects the activity of Ephexin4; however, this remains to be investigated.

In this study, we generated an Ephexin4 mutant (Ephexin4^E295A^) that abrogated the intermolecular interaction. RhoG was activated more efficiently by Ephexin4^E295A^ than by wild-type Ephexin4. This was because a higher level of RhoG bound to Ephexin4^E295A^ than to wild-type Ephexin4. In addition, the formation of membrane ruffles and engulfment of apoptotic cells were increased more by expression of Ephexin4^E295A^ than by expression of wild-type Ephexin4. Taken together, this study suggests that the intermolecular interaction of Ephexin4 autoinhibits its GEF activity by impeding binding of RhoG.

## 2. Materials and Methods

### 2.1. Plasmids and Reagents

All Ephexin4 mutants were generated by a polymerase chain reaction (PCR)-based strategy from the murine Ephexin4 cDNA (NM_001112744). Specifically, two point-mutated Ephexin4 (P271A and E295A) were generated using site-directed mutagenesis. GST-Elmo2^1-360^ was constructed by inserting the N-terminal part of the murine Elmo2 cDNA (NM_080287) into the pGEX-4T-2 vector. GFP-RhoG was used in the previous study [19]. The antibodies used in this study were anti-FLAG (Sigma, St. Louis, MO, USA, M2), anti-GFP (Santa Cruz Biotechnology, Dallas, TX, USA, FL), anti-GST (Santa Cruz Biotechnology, B-14), and anti-RhoG (Santa Cruz Biotechnology, 1F3 B3 E5).

### 2.2. Cell Culture and Transfections

293T cells were maintained in Dulbecco’s modified Eagle medium (DMEM) (Hyclone, Pittsburgh, PA, USA) supplemented with 10% FBS (Corning, Corning, NY, USA) and 1% penicillin/streptomycin/glutamine (Gibco). LR73 cells were maintained in alpha-MEM supplemented with 10% FBS and 1% penicillin/streptomycin/glutamine. 293T cells were transfected with calcium phosphate (Promega, Madison, WI, USA) and LR73 cells were transfected with Lipofectamine 2000 (Invitrogen, Carlsbad, CA, USA).

### 2.3. Immunoblotting and Immunoprecipitation

293T cells were transfected and incubated for 2 days. Then, cells were washed with ice-cold phosphate-buffered saline (PBS) and lysed with lysis buffer (50 mM Tris (pH 7.6), 150 mM NaCl, 10 mM NaPP, 10 mM NaF, 1 mM Na_3_VO_4_, 1% Triton X-100, 10 μg/mL pepstatin, 10 μg/mL leupeptin, 10 μg/mL AEBSF, and 10 μg/mL aprotinin). For immunoprecipitation assays, the lysates were incubated with the appropriate antibody-conjugated protein-A/G beads or Glutathione-Sepharose 4B beads for 2 h at 4 °C. After incubation, the beads were washed five times with wash buffer (20 mM HEPES (4-(2-hydroxyethyl)-1-piperazineethanesulfonic acid, pH 7.4), 150 mM NaCl, 5 mM NaF, 1 mM Na_3_VO_4_, 0.1% Triton X-100, 10% glycerol, 1 μg/mL pepstatin, 1 μg/mL leupeptin, 1 μg/mL AEBSF (4-(2-aminoethyl)benzenesulfonyl fluoride hydrochloride), and 1 μg/mL aprotinin). Lastly, proteins in the cell lysates and precipitation were assessed by immunoblotting. For RhoG-GEF binding assay, lysis/ wash buffer additionally contained 10 mM EDTA (Ethylenediaminetetraacetic acid). The band intensity of immunoblots were analyzed using ImageJ program.

### 2.4. Cross-Linking Assay

Chemical cross-linking assay was performed based on the manufacturer’s protocol. Briefly, the method is as follows. 293T cells were transfected with Ephexin4 or Ephexin4^E295A^ and the cells were rinsed twice with ice-cold PBS and harvested 2 days after transfection. The cells were resuspended in PBS (pH 8.0), incubated with DMSO (Dimethyl sulfoxide) or disuccinimidyl suberate (DSS) (0.1 mM, Thermo Scientific, Waltham, MA, USA) at room temperature for 30 min, and then terminated by adding Tris (pH 7.6, final conc. 20 mM). After that, the cells were lysed and proteins were detected by immunoblotting.

### 2.5. Purification of GST-ELMO2^1-360^

pGEX-4T-2/Elmo2^1-360^-transformed BL21(DE3) cells were induced with 1 mM IPTG (Isopropyl β-d-1-thiogalactopyranoside) overnight, harvested, resuspended in lysis buffer (100 mM Tris (pH7.4), 100 mM NaCl, 1 mM EDTA, 1 mg/mL lysozyme, 1 mM DTT (Dithiothreitol), 10 μg/mL pepstatin, 10 μg/mL leupeptin, 10 μg/mL aprotinin), and then lysed by sonication. The lysates were incubated with Glutathione-Sepharose 4B beads (GE Healthcare, Chicago, IL, USA) for 2 h at 4 °C. The beads were washed thoroughly and resuspended in wash buffer (50 mM Tris (pH 7.4), 150 mM NaCl, 1 mM DTT, 10 μg/mL pepstatin, 10 μg/mL leupeptin, 10 μg/mL aprotinin, 1% Triton X-100, 10% glycerol). Glycerol was added to the resuspended beads and stored at −80 °C until use.

### 2.6. Active RhoG Pull-Down Assay

293T cells were transfected with the indicated plasmids. 2 days after transfection, the cells were washed with ice-cold PBS and lysed. To precipitate active RhoG, the lysates were incubated with GST-ELMO2^1-360^ bound to Glutathione-Sepharose 4B beads for 1 h at 4 °C. After that, the beads were washed five times and precipitated proteins were detected by immunoblotting. The amount of RhoG precipitated with ELMO2^1-360^ was normalized to the total amount of RhoG in cell lysates for the comparison of GTP-bound RhoG levels. Active RhoG levels were quantified with ImageJ software

(ImageJ 1.49v, NIH, Bethesda, MD, USA).

### 2.7. Immunostaining

LR73 cells were plated on 18 mm Ø glass coverslips in a 12-well non-culture plate the day before transfection and the cells were transfected with the indicated plasmids. One day after transfection, cells were washed with PBS, fixed in 4% paraformaldehyde for 15 min, and permeabilized with 0.1% Triton X-100 for 5 min. Next, permeabilized cells were blocked with 1% BSA for 30 min and stained with Alexa Fluor 594-conjugated phalloidin (Life Technologies, Carlsbad, CA, USA) for 1 h at room temperature. After actin staining, nuclei were stained with Hoechst 33342 (Invitrogen, Carlsbad, CA, USA) and the coverslips were mounted on slides. Cell images were acquired on Zeiss Axio Imager D2 (Zeiss, Jena, German) or Olympus FV1000 SPD (Olympus, Tokyo, Japna).

### 2.8. Phagocytosis Assay

Phagocytosis assay was performed as reported previously [20]. Briefly, LR73 cells were transfected with the indicated plasmids. One day after transfection, apoptotic cell engulfment assays were performed as follows. Transfected cells were incubated with TAMRA (5-Carboxytetramethylrhodamine, Succinimidyl Ester)-labeled apoptotic thymocytes with 5% CO_2_ at 37 °C for 2 h. The ratio of phagocytes to apoptotic cells was 1:20. Next, LR73 cells were washed with cold PBS five times, trypsinized, resuspended, and analyzed by flow cytometry (BD FACS Canto II). The data acquired from flow cytometry were analyzed by the FLOWJO program. Double positive cells for GFP and TAMRA were considered as phagocytes engulfing apoptotic cells. MFI (mean fluorescence intensity) of engulfing phagocytes were evaluated using FLOWJO (FlowJo LLC, Ashland, OR, USA).

### 2.9. Statistical Analysis

All data are shown as mean ± standard deviation. Each experiment was performed independently at least three times, and the statistical significance of differences was evaluated by two-tailed *t* test using the GraphPad Prism 6 software (Prism 6, GraphPad Software, La Jolla, CA, USA). *p* < 0.05 was taken to indicate a significant difference.

## 3. Results

### 3.1. Generation of Ephexin4 Mutants that Abrogate the Intermolecular Interaction

**Figure 1:**
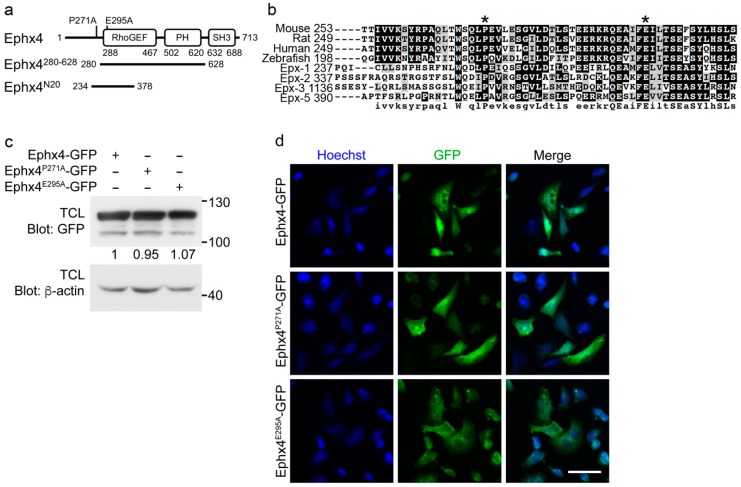
Generation of putative mutants of Ephexin4 that disrupt its intermolecular interaction. (**a**) Schematic diagram of the constructs used in this study. Ephx4, Ephexin4. (**b**) Amino acid sequence alignment of Ephexin proteins. Sequences were aligned using ClustalW and displayed using BoxShade. Asterisks indicate highly conserved residues in Ephexin proteins that were mutated in this study. (**c**) 293T cells were transfected with the indicated plasmids. Two days later, cells were lysed and proteins in the lysates were detected by immunoblotting. TCL, total cell lysate. *n* = 4. (**d**) LR73 cells were transfected with the indicated plasmids. GFP signals indicating expression of the transfected plasmids were observed by microscopy. Scale bar, 40 µm. *n* = 3. Images shown are representative of at least three independent experiments.

The functional domains of Ephexin family proteins are structurally conserved among homologs [18]. The N20 region, which corresponds to amino acids 234–378 of Ephexin4 and was originally identified as an Elmo-interacting fragment, mediates the intermolecular interaction of Ephexin4 by binding to the SH3 domain (Figure 1a) [16,19]. Therefore, we aligned the N20 regions of Ephexin4 homologs to identify which residues are essential for the intermolecular interaction. The proline residue at position 271 and the glutamate residue at position 295 in the N20 region are highly conserved (Figure 1b). To investigate the importance of these residues for the intermolecular interaction, we generated two Ephexin4 mutants, Ephexin4^P271A^ and Ephexin4^E295A^, in which the proline residue at position 271 or the glutamate residue at position 295 was substituted with alanine. To compare wild-type Ephexin4 and its mutants in an unbiased manner, we first evaluated whether the mutants had different expression levels or subcellular localization patterns. The expression levels of Ephexin4^P271A^ and Ephexin4^E295A^ were comparable with that of wild-type Ephexin4, although expression of the mutants was sometimes slightly lower than that of wild-type Ephexin4 (Figure 1c). Additionally, the subcellular localization patterns of the mutants did not differ from that of wild-type Ephexin4. GFP-tagged Ephexin4^P271A^ and Ephexin4^E295A^, as well as wild-type Ephexin4, were ubiquitously expressed in cells (Figure 1d). These data suggest that any effects of the mutants are not due to alteration of their expression levels or subcellular localization patterns.

### 3.2. The Glutamate Residue at Position 295 is Important for the Intermolecular Interaction of Ephexin4

**Figure 2:**
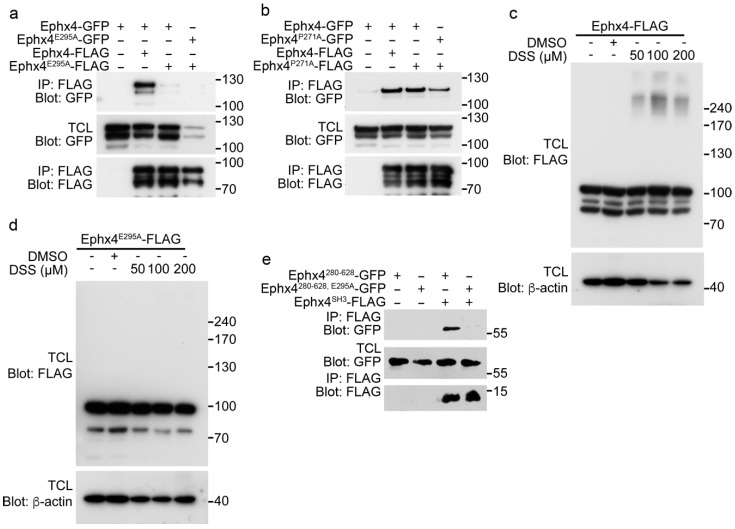
The E295A mutation disrupts the intermolecular interaction of Ephexin4. (**a**,**b**) 293T cells were transfected with the indicated plasmids and lysed 2 days later. Cell lysates were incubated with FLAG-conjugated agarose beads. Bead-bound proteins were separated by sodium dodecyl sulfate polyacrylamide gel electrophoresis (SDS-PAGE), transferred to a nitrocellulose membrane, and detected by immunoblotting. IP, immunoprecipitation. *n* = 3. (**c**,**d**) 293T cells were transfected with Ephexin4 (**c**) or Ephexin4^E295A^ (**d**). Two days later, cells were harvested, resuspended in PBS, and incubated with 0.1 mM DSS for 30 min. Thereafter, cells were lysed and proteins were detected by immunoblotting. *n* = 4. (**e**) 293T cells were transfected with the indicated plasmids. Two days later, cells were lysed and cell lysates were incubated with FLAG-conjugated agarose beads. Bead-bound proteins were detected by immunoblotting. *n* = 3. Images shown are representative of at least three independent experiments.

Next, we investigated whether these mutations of Ephexin4 affect its intermolecular interaction using two different approaches. First, wild-type Ephexin4 and its mutants were expressed in 293T cells and immunoprecipitation assays were performed. Remarkably, neither GFP-tagged wild-type Ephexin4 nor Ephexin4^E295A^ co-immunoprecipitated with FLAG-tagged Ephexin4^E295A^, whereas GFP-tagged wild-type Ephexin4 co-immunoprecipitated with FLAG-tagged wild-type Ephexin4 (Figure 2a). However, Ephexin4^P271A^ co-precipitated with wild-type Ephexin4 and Ephexin4^P271A^, although a slightly lower level of GFP-tagged Ephexin4^P271A^ than wild-type Ephexin4 co-immunoprecipitated with FLAG-tagged Ephexin4^P271A^ (Figure 2b). These data suggest that the glutamate residue at position 295 is important for the intermolecular interaction of Ephexin4. Second, we further investigated the importance of this glutamate residue for the intermolecular interaction by performing a crosslinking assay. Cells expressing wild-type Ephexin4 were treated with DSS, a crosslinker that links amine groups of proteins and forms amide bonds, and then Ephexin4 was detected by immunoblotting. New bands approximately 3-fold larger than Ephexin4 (250 kDa) were detected above the normal Ephexin4 bands when cells expressing wild-type Ephexin4 were treated with DSS, but not when they were treated with the DMSO control (Figure 2c). This indicates that wild-type Ephexin4 molecules are located close enough for crosslinking to occur. By contrast, new bands were not observed at the same size when cells expressing Ephexin4^E295A^ were treated with DSS (Figure 2d), suggesting that Ephexin4^E295A^ molecules are not positioned close enough for crosslinking to occur and thus that this mutation disrupts the intermolecular interaction of Ephexin4.

The intermolecular interaction of Ephexin4 is mediated by its SH3 domain, which interacts with the region containing amino acids 280–628 [19]. Thus, we investigated whether the disrupted intermolecular interaction of Ephexin4 is due to alteration of the association between the SH3 domain and amino acids 280–628. Intriguingly, the E295A mutation completely abolished the interaction of the SH3 domain with amino acids 280–628 (Figure 2e). These data indicate that inhibition of the association of the SH3 domain with amino acids 280–628 abolishes the intermolecular interaction of Ephexin4.

### 3.3. Augmented Binding of RhoG to Ephexin4^E295A^ Results in Notable RhoG Activation

**Figure 3:**
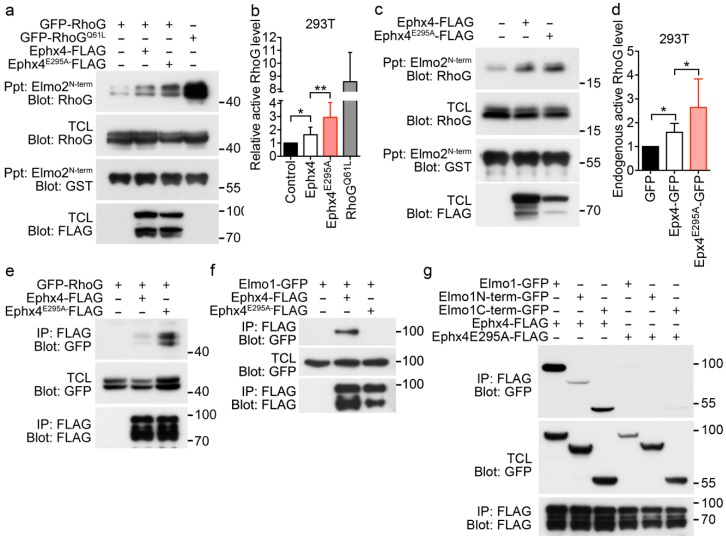
RhoG is activated more by Ephexin4^E295A^ than by wild-type Ephexin4. (**a**) 293T cells were transfected with the indicated plasmids and lysed 2 days later. Cell lysates were incubated with GST-Elmo2^N-term^ bound to glutathione-sepharose beads. Bead-bound proteins were separated by SDS-PAGE, transferred to a nitrocellulose membrane, and detected by immunoblotting. *n* = 4. (**b**) Quantification of active RhoG levels in five independent experiments performed as described in (**a**). (**c**,**d**) After transfection of 293T cells with wild-type Ephexin4 or Ephexin4^E295A^, endogenous GTP-bound RhoG was detected (**c**) and quantified (**d**). *n* = 3. (**e**–**g**) 293T cells were transfected with the indicated plasmids. Two days later, cells were lysed and cell lysates were incubated with FLAG-conjugated agarose beads. Bead-bound proteins were detected by immunoblotting. *n* = 3. Data are shown as the mean ± standard deviation and are representative of at least three independent experiments. * *p* < 0.05, ** *p* < 0.01.

GEFs activate small GTPases by stimulating release of GDP to allow binding of GTP. The capability of GEFs to exchange GDP with GTP in GTPases is regulated in various ways. One such regulatory mechanism is autoinhibition, which is usually mediated by steric hinderance [21,22,23,24]. Thus, we investigated whether the intermolecular interaction of Ephexin4 affects its GEF activity. To this end, we compared activation of RhoG by wild-type Ephexin4 with that by Ephexin4^E295A^. We measured the levels of active RhoG by performing a pulldown assay using Elmo2^N-term^, which only precipitated GTP-bound RhoG. The level of active RhoG was 1.6-fold higher in cells expressing wild-type Ephexin4 than in control cells. Interestingly, the level of active RhoG was increased even more in cells expressing Ephexin4^E295A^. The level of active RhoG was 2.9-fold higher in cells expressing Ephexin4^E295A^ than in control cells (Figure 3a,b). In addition, Ephexin4^E295A^ increased activation of endogenous RhoG. The level of active RhoG was increased by 1.6-fold in cells expressing wild-type Ephexin4 and by 2.6-fold in cells expressing Ephexin4^E295A^ (Figure 3c,d), suggesting that RhoG is activated more by Ephexin4^E295A^ than by wild-type Ephexin4.

Next, we investigated whether the notable activation of RhoG by Ephexin4^E295A^ is due to increased binding of RhoG. To this end, we measured the amounts of RhoG bound to wild-type Ephexin4 and Ephexin4^E295A^. A larger amount of RhoG co-immunoprecipitated with Ephexin4^E295A^ than with wild-type Ephexin4 (Figure 3e). These data suggest that RhoG binds more readily to Ephexin4^E295A^ than to wild-type Ephexin4 and that the intermolecular interaction of Ephexin4 impedes binding of RhoG.

### 3.4. Elmo1 is Dispensible for the Notable RhoG Activation by Ephexin4^E295A^

The biochemical interaction of Ephexin4 with Elmo augments binding of RhoG to Ephexin4 [19]. Thus, the prominent activation of RhoG by Ephexin4^E295A^ in comparison with wild-type Ephexin4 may be because the interaction between Ephexin4^E295A^ and Elmo is stronger than that between wild-type Ephexin4 and Elmo. To investigate this possibility, we compared the interactions of Ephexin4^E295A^ and wild-type Ephexin4 with Elmo. Unexpectedly, wild-type Ephexin4 co-immunoprecipitated with Elmo1, while Ephexin4^E295A^ did not (Figure 3f). Elmo1 contains two Ephexin4-binding sites, one in the N-terminal region and the other in the C-terminal region. Therefore, we further investigated whether the E295A mutation abrogates both these interactions of Elmo1 with Ephexin4. Wild-type Ephexin4 strongly interacted with full-length Elmo1 and the N-terminal and C-terminal fragments, whereas Ephexin4^E295A^ did not interact with any of these proteins (Figure 3g). These data indicate that the E295A mutation of Ephexin4 completely abolishes its interactions with the N-terminal and C-terminal regions of Elmo1 and that the notable activation of RhoG by Ephexin4^E295A^ is due to disruption of the intermolecular interaction, not to the cooperative effects of Elmo1 on RhoG activation.

### 3.5. Ephexin4^E295A^ Induces Phagocytosis of Apoptotic Cells and Membrane Ruffle Formation to a Greater Extent than Wild-Type Ephexin4

**Figure 4:**
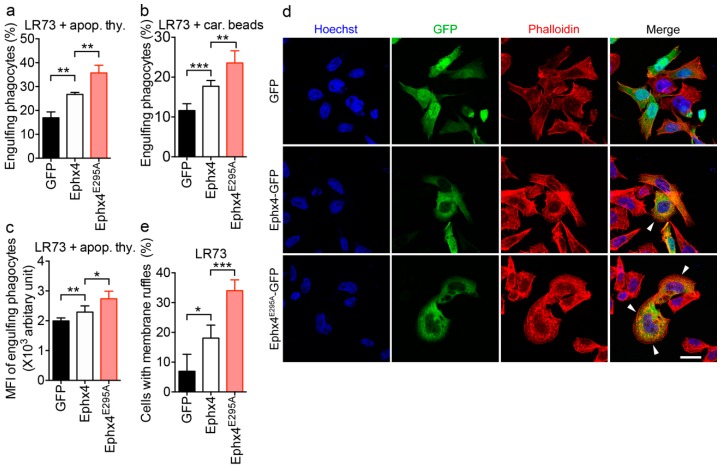
Ephexin4^E295A^ induces RhoG-mediated processes to a greater extent than wild-type Ephexin4. (**a**–**c**) LR73 cells were transfected with the indicated plasmids and then incubated with TAMRA (5-Carboxytetramethylrhodamine, Succinimidyl Ester)-stained apoptotic thymocytes (**a**,**c**) or 2 µm carboxylate-modified beads (red fluorescence, (**b**)) for 2 h. Thereafter, cells were extensively washed with ice-cold phosphate-buffered saline (PBS) five times, trypsinized, and analyzed by flow cytometry. Cells positive for GFP and red fluorescence were considered to be phagocytes engulfing apoptotic cells (**a**,**c**) and carboxylate beads (**b**), respectively. *n* = 4. (**d**,**e**) LR73 cells were transfected with the indicated plasmids. One day later, cells were stained with Alexa Fluor 594-conjugated phalloidin and then with Hoechst 33342. Images were acquired by microscopy (**d**). At least 100 transfected cells were counted in randomly selected areas. Arrowheads indicate membrane ruffles. Scale bar, 20 µm. The percentage of GFP-positive cells with membrane ruffles was calculated (**e**). *n* = 3. Data are shown as the mean ± standard deviation and are representative of at least three independent experiments. * *p* < 0.05, ** *p* < 0.01, *** *p* < 0.001.

RhoG is involved in a variety of cellular processes. For example, RhoG activates Rac, which induces rearrangement of the actin cytoskeleton and thereby promotes phagocytosis of apoptotic cells [16,25,26,27,28]. Thus, we investigated whether the notable activation of RhoG by Ephexin4^E295A^ affects the phagocytosis of apoptotic cells and formation of membrane ruffles which are forms on a motile cell surface that contains a meshwork of newly polymerized actin filaments. We first evaluated engulfment of apoptotic cells and carboxylate-modified beads, which mimic the negative surface charge of apoptotic cells, by LR73 cells exogenously expressing Ephexin4^E295A^ or wild-type Ephexin4. Wild-type Ephexin4 enhanced phagocytosis of apoptotic cells and beads, as reported previously [19]. Intriguingly, higher percentages of Ephexin4^E295A^-expressing cells than wild-type Ephexin4-expressing cells engulfed apoptotic cells and beads (Figure 4a,b). Moreover, the mean fluorescence intensity (MFI), indicative of the relative number of ingested targets per phagocyte, was higher in Ephexin4^E295A^-expressing cells than in wild-type Ephexin4-expressing cells (Figure 4c).

In addition, we compared membrane ruffle formation induced by Ephexin4^E295A^ with that induced by wild-type Ephexin4. Cells expressing wild-type Ephexin4 contained more membrane ruffles than cells expressing GFP. However, the number of membrane ruffles per cell was higher in Ephexin4^E295A^-expressing cells than in wild-type Ephexin4-expressing cells, and a higher percentage of Ephexin4^E295A^-expressing cells than wild-type Ephexin4-expressing cells contained membrane ruffles (Figure 4d,e). This indicates that the effects of Ephexin4^E295A^ on RhoG-related cellular processes are greater than those of wild-type Ephexin4. Taken together, these data suggest that the intermolecular interaction of Ephexin4 autoinhibits its GEF activity by impeding binding of RhoG.

## 4. Discussion

Ephexin4 is a member of the Ephexin subfamily of GEFs, which directly interact with EphA receptors. Ephexin4 associates with EphA2 and mediates cell migration by activating RhoG. EphA2 binds to Ephexin4 and induces activation of Ephexin4 and RhoG in response to epidermal growth factor stimulation [8], which suggests that the GEF activity of Ephexin4 must be suppressed in the basal state. This study reports that Ephexin4 is autoinhibited via its intermolecular interaction and that disruption of this interaction enhances the GEF activity of Ephexin4. Accordingly, it is plausible that the intermolecular interaction of Ephexin4 is maintained when it is not associated with EphA2, but is disrupted when Ephexin4 associates with EphA2, allowing binding of RhoG to the DH domain of Ephexin4. Elmo may participate in this process via its association with Ephexin4, resulting in synergistic activation of RhoG. It will be interesting to investigate if and how EphA2, Ephexin4, and Elmo associate with each other and affect activation of RhoG. Crystallography will help to determine the stoichiometry of these proteins.

This study suggests that disruption of the intermolecular interaction of Ephexin4 increases its GEF activity by allowing binding of RhoG to the DH domain. Thus, Ephexin4^E295A^ is a constitutively active form of Ephexin4. Other mutants that disrupt the intermolecular interaction of Ephexin4 and thereby allow binding of RhoG may also be constitutively active. In this study, 293T cells were mainly used to evaluates the effects of Ephexi4 and Ephexin4^E295A^. In addition to 293T cells, a human colon cancer cell line (HCT116) was also used to validate phenomena caused by Ephexin4^E295A^ in a different cell type. Although there was a difference in degree, Ephexin4^E295A^ abrogated the homotypic intermolecular interaction of Ephexin4 and failed to form an oligomer in HCT116 as well (data not shown). Therefore, the effects of Ephexin4^E295A^ are not cell-type specific but general events. Furthermore, Ephexin4 is involved in cancer cell migration; therefore, it would be interesting to investigate whether a mutation of Ephexin4 that abrogates its intermolecular interaction occurs in any metastatic cancer.

It is reported that RhoG activation promotes neural progenitor cell proliferation [29]. Thus, we also tested whether wild-type Ephexin4 or its mutants (Ephexin4^E295A^ and Ephexin4^ΔSH3^) could promote cell proliferation in 293T cells. None of them enhanced cell proliferation (data not shown), suggesting that cell proliferation promoted by RhoG could be specific in certain cell types. Accordingly, although RhoG activation by Ephexin4 is a global event, the effect of RhoG activation on downstream cellular events might be cell- or tissue-specific.

Unexpectedly we found that the E295A mutation disrupted the interaction of Ephexin4 with Elmo1. In a previous study, we reported that Ephexin4 and Elmo1 associate via interactions between the SH3 domain of Ephexin4 and the C-terminal region of Elmo1, and between the N20 region of Ephexin4 and the N-terminal region of Elmo1 [19]. The E295A mutation of Ephexin4 is located in the N20 region. Thus, while this mutation was anticipated to perturb the interaction between the N20 region of Ephexin4 and the N-terminal region of Elmo1, it was not expected to disrupt the interaction between the SH3 domain of Ephexin4 and the C-terminal region of Elmo1. It is possible that the E295A mutation induces a conformational change of Ephexin4 that masks the binding site for the C-terminal region of Elmo1. Thus, the E295A mutation does not weaken the interaction with Elmo1 but completely abolishes it.

The Ephexin subfamily of GEFs has five members. All Ephexin proteins, with the exception of Ephexin5, have a long *N*-terminal domain and a tandem DH-PH domain followed by a SH3 domain, suggesting that other members of the Ephexin subfamily are regulated in a similar manner to Ephexin4. We found that Ephexin1 and Ephexin2 also have intermolecular interactions (unpublished data), which supports this idea. This would be an interesting topic to investigate in the future.

Collectively, this study suggests that Ephexin4 is autoinhibited via its intermolecular interaction, which leads to steric hinderance and impedes binding of RhoG. Given that Ephexin4 is involved in the migration of cancer cells and clearance of apoptotic cells, the findings of the study can be used to develop therapeutics for cancers and diseases caused by defects in the clearance of apoptotic cells.
